# Pressure Effect of the Vibrational and Thermodynamic Properties of Chalcopyrite-Type Compound AgGaS_2_: A First-Principles Investigation

**DOI:** 10.3390/ma11122370

**Published:** 2018-11-26

**Authors:** Jianhui Yang, Qiang Fan, You Yu, Weibin Zhang

**Affiliations:** 1 School of Physics and Electronic Engineering, Leshan Normal University, Leshan 614004, China; yjh20021220@foxmail.com; 2 College of Optoelectronic Technology, Chengdu University of Information Technology, Chengdu 610225, China; yy2012@cuit.edu.cn; 3 School of Physics and Optoelectronic Engineering, Yangtze University, Jingzhou 434023, China; zmrright@163.com; 4 Department of Physics, Dongguk University, Seoul 04620, Korea

**Keywords:** Ag-based chalcopyrite type compound, first principles, lattice dynamics, thermodynamic properties

## Abstract

To explore the structural, vibrational, and thermodynamic properties of the chalcopyrite-type compound AgGaS_2_ under pressure, we applied hydrostatic pressure to the relaxed compound based on the first principles calculation and quasi-harmonic approximation. The structural parameters, including lattice constants and bond lengths decrease monotonically with the increasing pressure. The phonon dispersion curves under various pressures reveal the structural phase transition of chalcopyrite-type compound AgGaS_2_ at about 4 GPa. The intrinsic mechanism of thermal conductivity for the chalcopyrite-type compound AgGaS_2_ has been shown with phonon anharmonicity. The frequencies of the optical phonons at the center point Γ of the first Brillouin zone were calculated with the longitudinal optical–transverse optical (LO–TO) splitting mode. The dependence of the frequencies of the optical phonons on the pressure provides the information for the Raman spectroscopic study under high pressure. The pressure dependence of the Grüneisen parameters indicates that the instability of chalcopyrite-type compound AgGaS_2_ is associated with the softening of the acoustic phonon modes at around the center point Γ. The thermal conductivity for chalcopyrite-type compound AgGaS_2_ could be reduced by applying external pressure. The various thermodynamic properties, such as the Helmholtz free energy, entropy, and heat capacity, at different temperatures and pressures were discussed and analyzed based on the phonon properties.

## 1. Introduction

I-III-VI_2_ ternary chalcopyrite type compounds have attracted attention as extracting candidates in non-linear optic [1,2,3], novel spintronic [4], and thermoelectric [5,6,7] devices, as well as in thin film solar cells [8,9,10]. AgGaS_2_, as a typical member of I-III-VI_2_ ternary chalcopyrite-type compounds, has attracted interest from researchers [11,12,13]. The low thermal conductivity (1.4 W/m K) [14] makes chalcopyrite type compound AgGaS_2_ known as a potential thermoelectric material. Understanding the physical mechanism of the thermal conductivity of AgGaS_2_ is necessary to discuss how to reduce the thermal conductivity and promote the application in a thermoelectric device. The investigations of lattice dynamic and thermodynamic properties have great practical significance for the engineering application of materials. Holah et al. presented the lattice dynamic properties of chalcopyrite-type compound AgGaS_2_ in an experiment for the first time in 1974 [15]. Neumann et al. measured the heat capacity and Grüneisen parameters of the chalcopyrite-type compound AgGaS_2_ [16]. Besides experimental research, the lattice dynamic and thermodynamic properties of the chalcopyrite-type compound AgGaS_2_ have also attracted the attention of theoretical researchers for its potential application. The phonon dispersion relations were performed by Łażewski et al. using first principles within local density approximation [17]. Wei et al. investigated the anharmonicity of the acoustic phonon of AgGaS_2_ using first principles under the three-phonon Umklapp process [18]. Recently, Kushwaha et al. calculated the lattice dynamic properties of AgGaS_2_, including Raman and infrared inactive modes, using rigid-ion model [19]. Nevertheless, most of the lattice dynamic and thermodynamic investigations of AgGaS_2_ were carried out at 0 pressure.

It is well known that the electronic and geometrical structures will undergo changes under external pressure, which induces novel properties and have many wide applications, such as pressure-induced phase transitions [20,21], derived-spin crossover transitions [22], enhanced superconductivity [23,24], and improved optical, thermodynamic, and thermoelectric performances [25,26,27]. The pressure-inducing scheme is straightforward to improve the non-linear optical properties of AgGaS_2_ as calculated by Li et al. [28] It is important to note that the chalcopyrite structure tends to deform under pressure. Several experimental investigations revealed that the pressure-induced phase transition occurs from space group symmetry of $I\bar{4}2d$ to *Cc* at about 4.2 GPa [29,30]. However, no theoretical research has yet been carried out to access the pressure-induced phase transition based on the crystal lattice dynamics. It is worth investigating which vibrational modes and regions are related to phase transitions under pressure. On the other hand, the intrinsic mechanism of the thermal conductivity of AgGaS_2_ is still not clearly known. Besides, the pressure dependence of thermodynamic properties is a valuable research to clarify the influence of pressure on the chalcopyrite-type compound AgGaS_2_.

The purpose of our work is to investigate the dynamic stability, the vibrational and thermodynamic properties of the chalcopyrite-type compound AgGaS_2_ under different hydrostatic pressures by using first-principles. The dynamic stability was verified by analyzing the corresponding phonon spectrum under various pressures. The vibrational properties, including the effect of longitudinal optical–transverse optical (LO–TO) splitting, were investigated using the finite displacement method. The Grüneisen parameters and thermodynamic properties were obtained by using a quasi-harmonic approximation method.

## 2. Computational Scheme

The structural optimization and self-consistent calculations were performed using Vienna Ab-initio Simulation Package (VASP) code [31] within the framework of density functional theory (DFT). The Perdew–Bruke–Ernzerhof (PBE) functional for the generalized gradient approximation (GGA) was used to describe the exchange correlation potential. The projector augmented wave pseudopotentials with a 400 eV cut-off energy were employed to describe the interaction between the electrons and ions. The valence states of the atoms of Ag, Ga, and S were chosen as 4d^10^5s^1^, 4s^2^4p^1^, and 3s^2^sp^4^, respectively. The structure relaxation was performed until all forces acting on each atom were smaller than 10^−5^ eV/Å and the total energy change between 2 self-consistent steps was smaller than 10^−8^ eV. The first Brillouin zone of a unit cell was sampled by a 3 × 5 × 4 k-mesh scheme with Γ central for structural optimization.

We used the finite-displacement method within the framework of the force constants as implemented in PHONONPY code to determine the vibrational properties [32]. The necessary force constants of the supercell were computed in real space by using the finite displacement method with the help of the VASP code. The 2 × 2 × 2 supercell of the unit cell and a 3 × 3 × 3 k-mesh were used to calculate the force constants, which were confirmed to provide converged results. Based on our knowledge of group theory, we also analyzed the vibration modes of the center point Γ. The Grüneisen parameters and thermodynamic properties, such as the Helmholtz-free energy, entropy, and heat capacity, were calculated from the phonon density of states (DOS) as a function of the frequencies. The relevant calculation scheme was carried out in the framework of the quasi-harmonic approximation (QHA), in which the phonon frequencies are volume-dependent.

## 3. Results and Discussions

### 3.1. Structural Optimization

**Figure 1:**
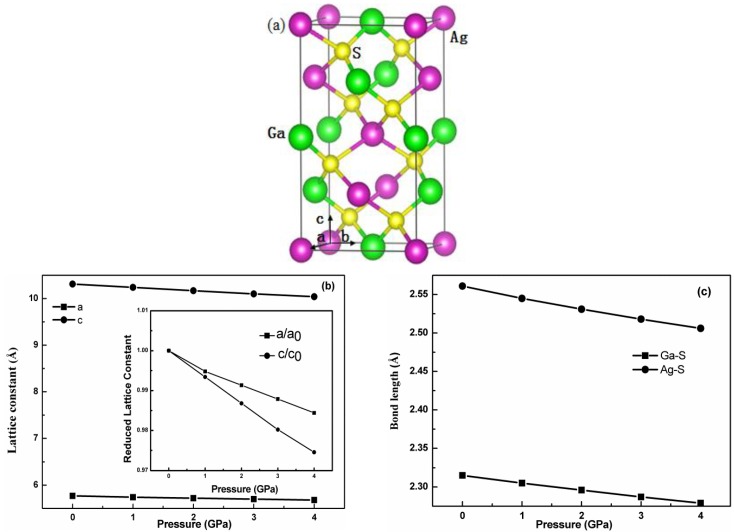
(**a**) Crystal structure, (**b**) calculated lattice constants, and (**c**) bond lengths of Ga-S and Ag-S for the chalcopyrite-type compound AgGaS_2_ as a function of the hydrostatic pressure.

The chalcopyrite-type compound AgGaS_2_ belongs to the $I\bar{4}2d$ space group, with the Ag, Ga, and S atoms occupying the Wyckoff sites of 4a, 4b, and 8d, represented as purple, green, and yellow balls in Figure 1a, respectively. Based on the theoretically predicted structure of the chalcopyrite-type compound AgGaS_2_ [33], the parameter constants and atomic coordinates under different pressures were fully carried out until the total energy and force acting on atom reach convergence.

The optimized lattice constants for chalcopyrite type compound AgGaS_2_ under 0 pressure are a = b = 5.77 Å, and c = 10.31 Å, and these are in very good agreement with the experimental data [34]. The difference of the lattice constants between this work and other theoretical computations is almost less than 3% [35,36]. The respective bond lengths of Ag-S and Ga-S for chalcopyrite type compound AgGaS_2_ under 0 pressure are 2.561 and 2.315 Å, which is close to the previous prediction [37]. Figure 1b shows the dependence of the lattice constants on the pressure up to 4 GPa. We found that the lattice constants gradually decrease with the pressure increase. The variation of the lattice constants (a and c) presents anisotropic property when external pressure is applied. To display the anisotropic character more clearly, the reduced lattice constants are plotted together as the insert in Figure 1b, where a_0_ and c_0_ are the lattice constants at 0 pressure. The c/c_0_ drops faster than the a/a_0_ with pressure, which demonstrates that chalcopyrite-type compound AgGaS_2_ is easily compressed along the c direction; the previous experimental studies showed exactly the same conclusion [38,39]. The bond length is one of the important structural parameters. To further clarify the effect of the pressure on the structure, the hydrostatic pressure dependence on the bond length is shown in Figure 1c. It is worth noting that the bond lengths of Ag-S and Ga-S for chalcopyrite-type compound AgGaS_2_ decrease gradually with the increasing hydrostatic pressure in the range of the pressure studied. Moreover, the bond length of Ag-S is more sensitive than that of Ga-S with the increasing hydrostatic pressure, which is consistent with experimental prediction by single-crystal X-ray diffraction [30]. As proved later, the broken tetrahedron structure induces structural phase transition. The AgS_4_ tetrahedron undergoes a slightly larger geometrical deformation and could be broken more easily than the GaS_4_ tetrahedron under pressure.

### 3.2. Vibrational Properties

**Figure 2:**
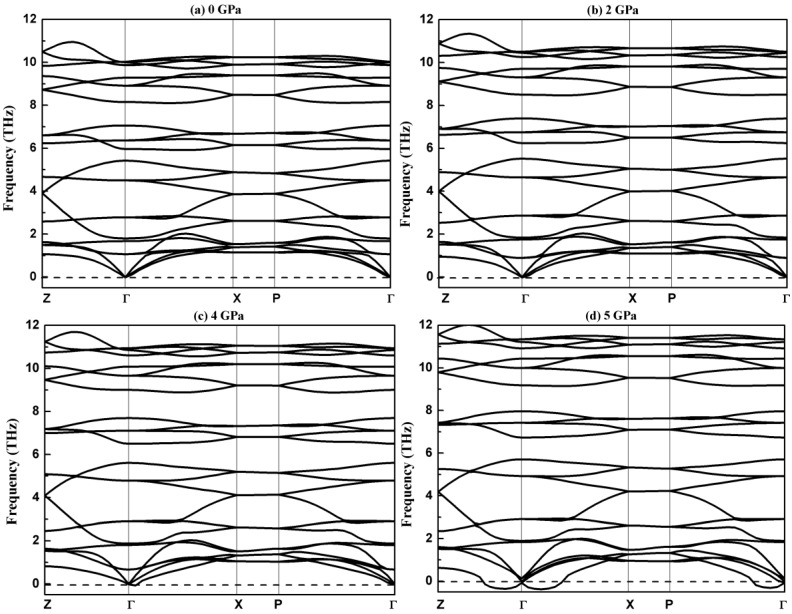
Phonon spectrums along the high-symmetry directions of AgGaS_2_ at different hydrostatic pressures: (**a**) 0 Gpa; (**b**) 2 Gpa; (**c**) 4 Gpa; (**d**) 5 Gpa.

The phonon characteristics directly reflect dynamic stability and play a vital role in the physical properties of materials. We now turn to the vibrational properties of chalcopyrite-type compound AgGaS_2_. Figure 2 exhibits the computational phonon spectrums along the high symmetry line Z(1/2,1/2,−1/2)→Γ(0,0,0)→X(0,0,1/2)→P(1/4,1/4,1/4)→Γ(0,0,0) in the first Brillouin zone at different pressures.

Figure 2 shows there are no imaginary frequencies in the whole Brillouin zone below 4 GPa, which indicates that AgGaS_2_ with the $I\bar{4}2d$ structure is dynamically stable below 4 GPa. The phonon spectrum under 5 Gpa shows an obvious imaginary frequency near the center point Γ. The appearance of the imaginary frequency is a sure sign of a dynamic instability at the related pressure; the appearance of an imaginary frequency is related to the phase transition. The imaginary frequency comes from the *E* vibrational mode as confirmed from analysis of the vibrational mode. The single-crystal X-ray diffraction shows that the chalcopyrite type compound AgGaS_2_ in a tetragonal phase with space group of $I\bar{4}2d$ can change to a monoclinic phase with a space group of *Cc* at 4.2 GPa [30]. The purpose of this work is to reveal the pressure effect on the vibrational and thermodynamic properties of AgGaS_2_ in the tetragonal phase. The vibrational properties less than 4 GPa of chalcopyrite type compound AgGaS_2_ are discussed below.

**Figure 3:**
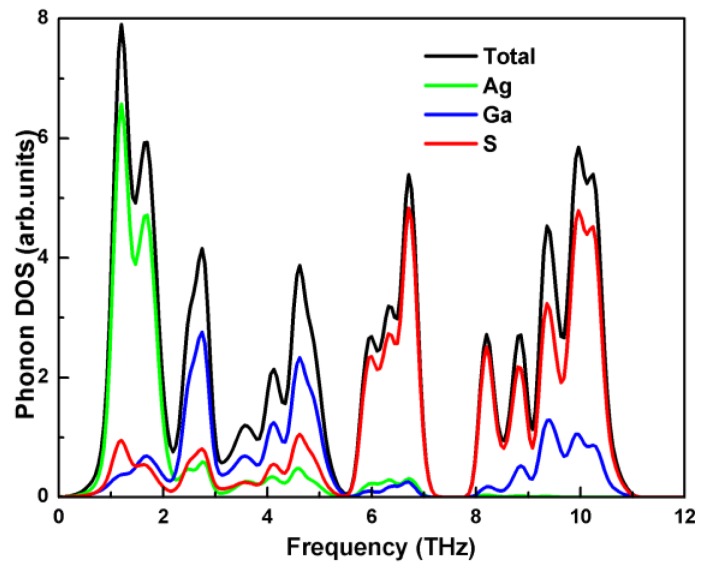
Total and partial phonon densities of the states (DOS) of AgGaS_2_ at 0 GPa.

Because there are 8 atoms in the unit cell of chalcopyrite type compound AgGaS_2_, the phonon spectrum includes 24 branches (3 acoustic and 21 optical branches). The phonon spectrum is divided into 3 energy intervals; the 3 acoustic branches are linear in wave vector ***q*** for a small ***q***. The contribution of optical branches to thermal conductivity is not considered with their small group velocities, seeing from the flat distribution in Figure 2. The optical branches overlap with the acoustic branches in low frequency, which benefits to the low conductivity of AgGaS_2_ as confirmed in other chalcopyrite type compound CuInTe_2_ [40]. The flat phonon branches along the X–P direction indicate the intracellular interactions are stronger than those between the cells in this direction. We note that all modes along the X–P direction are double-degenerated. As shown in Figure 2, the frequency of the phonon increases with the increase of pressure. The fundamental cause of this phenomenon is that the bond lengths of Ag-S and Ga-S become shorter with the increase of pressure. The shortening bond lengths lead to larger force constants, which benefits higher vibration frequencies. We investigated the vibrational property further to explore the contribution of the atoms to the phonon spectrum; the total and partial phonon densities of chalcopyrite type compound AgGaS_2_ at 0 GPa are plotted in Figure 3.

The partial phonon DOS in Figure 3 shows that the vibration of the Ag atom governs the acoustic branches. The vibration of the S atom contributes to the optical branches in the high energy region for the light mass. The main contributor to the optical branches in the frequency range from 2.2 THz to 5.5 THz is the Ga atom. Therefore, it is a scheme to reduce the thermal conductivity by inducing impurity scattering at Ag atom position.

Twenty-four dispersion curves mean there are 24 normal vibrational modes at the center of the Brillouin zone (Γ point), which can be explicated by group theory. The $I\bar{4}2d$ space group belongs to the *D*_2*d*_ point group. Based on the group theory, the *D*_2*d*_ point group has 4 one-fold-degenerate (*A*_1_, *A*_2_, *B*_1_, *B*_2_) and 1 two-fold-degenerate (*E*) irreducible representations. The Ag and Ga atoms are located at the S_4_ symmetric crystal positions, and the vibration modes can be described as simple motion parallel to the 3 crystal axes. The irreducible representation of the lattice vibrations for the Ag and Ga atoms are represented as:

(1) $$\Gamma_{Ag}=\Gamma_{Ga}=B_{1}+B_{2}+2E.$$

The sulfur atom is located on the *C*_2_ symmetric position, whose vibration modes can be expressed as the following irreducible representation:

(2) $$\Gamma_{s}=A_{1}+2A_{2}+1B_{1}+2B_{2}+3E.$$

The *A*_1_ and *A*_2_ vibrational modes only involve the displacement of the sulfur atom. The *B*_1_, *B*_2_, and *E* vibrational modes include the displacement of all atoms. The corresponding irreducible representation of the acoustic and optical vibrational modes at center point Γ can be represented as:

(3) $$\Gamma_{aco}=B_{2}+E.$$

(4) $$\Gamma_{opt}=A_{1}+2A_{2}+3B_{1}+3B_{2}+6E.$$

The 3 acoustic vibrational modes are neither infrared nor Raman active. For 21 optical vibrational modes, *A*_1_, *B*_1_, *B*_2_, and *E* are Raman active; furthermore, *E* and *B*_2_ are also infrared active. The *A*_2_ vibrational mode is called silent vibrational mode, since it is neither infrared nor Raman active. There are 13 Raman active vibrational modes, 9 infrared active vibrational modes and 2 silent vibrational modes at center point Γ. We took into account the long-range polarization interactions, so the infrared polar modes *B*_2_ and *E* were split into the longitudinal-optical (LO) and transverse-optical (TO) components. The LO–TO splitting depends on the values of the macroscopic dielectric constant and Born effective charges. As the polarization field increases the restoring force of the atomic displacement, the frequency of the LO mode increases. The splitting of the *B*_2_ vibration mode is parallel to the c axis, while the splitting of the *E* vibration mode is perpendicular to the c axis. The calculated frequencies of the optical phonons, including LO–TO splitting from the *B*_2_ and *E* modes at the center point Γ under different hydrostatic pressures, are listed in Table 1. The frequencies for the LO and TO components are separated with a slash in Table 1. The first column represents the irreducible representations of vibrational modes, abbreviated as “Irreps”.

As noted by Ohrendorf [42], the quality of a single crystal, including its impurity and lattice defects, leads to a difference of the vibrational wavenumbers in the different measurements in some cases. Based on reliable experimental data, Harran et al. estimated the values of the vibrational frequencies of AgGaS_2_ [42]. Recently, Kushwaha et al. [19], employed the rigid-ion model with the help of the reliable phonon mode from the literature [42] as input data, calculated the vibration modes of AgGaS_2_. The vibrational frequencies from the rigid-ion model are in good agreement with the experimental data, as shown in Table 1. The calculated results from the DFT frame are about 9% lower than the experimental results, which is a well-known underestimation of the phonon frequency by using the PBE functional [43,44,45]. To study the behavior of the phonon structure in greater detail, we analyzed the pressure dependence of the phonon frequency at center point Γ. We were surprised to find that the frequencies of all modes expect the lowest *E* mode increase with the increasing pressure; the tendency was discovered in the pressure dependence of phonon frequency of ZnGeP_2_ with the chalcopyrite structure by Raman scattering measurement [46]. The significant downtrend for the frequency of the lowest *E* mode with the increase in pressure means the soft *E* mode may influence the structural stability; this same phenomenon was found in the pressure-induced phase transition of SnSe crystal [47].

**Figure 4:**
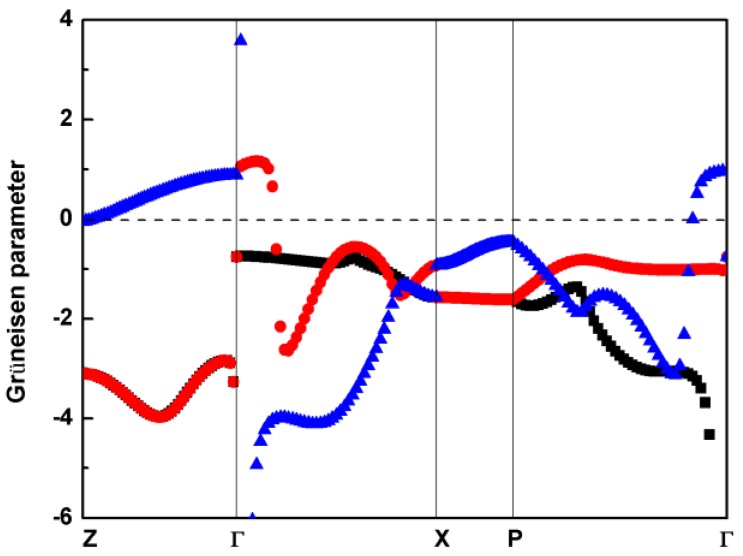
Mode Grüneisen parameter of acoustic phonons for AgGaS_2_ along high symmetry points at 0 GPa.

The phonon spectrum figures out the harmonicity of interatomic force constant. The thermal conductivity, however, is determined in the term of phonon anharmonicity, characterized by the Grüneisen parameter. The Grüneisen parameter can be described with the relative change of a phonon frequency due to the change of the cell volume. The value of the Grüneisen parameter represents the thermal expansion behavior of the materials, too. The mode of the Grüneisen parameter at the wave vector ***q*** and band index *j* is given in the following equation:

(5) $$\gamma_{qj}=-\frac{dln\omega_{qj}}{dlnV}=-\frac{V}{\omega_{qj}}\frac{\partial \omega_{qj}}{\partial V},$$

where $\omega_{qj}$ is the frequency of the *j*th phonon mode at wave vector ***q***, and *V* is the volume. To obtain the shift of the phonon frequency caused by the volume change, it is necessary to consider 3 phonon calculations under 3 different volumes. The 3 volumes are the equilibrium volume, a volume slightly smaller than the equilibrium volume, and a volume slightly larger than the equilibrium volume. In our work, we compressed and expanded the equilibrium volume of AgGaS_2_ by 0.3%. The calculated mode Grüneisen parameters of the 3 acoustic branches under 0 pressure along the high symmetry points are shown in Figure 4, in which the black and red symbols denote the transverse acoustic (TA) mode, and the longitudinal acoustic (LA) mode is represented with the blue symbol. 

As shown in Figure 4, most of the mode Grüneisen parameters are negative, which is generally consistent with previous first-principles calculational results [18]. The negative Grüneisen parameters imply a negative coefficient of the thermal expansion, coming from the tetrahedral structures of Ag-S and Ga-S in the crystal structure, as proved in other tetrahedral materials [48]. We found that the Grüneisen parameters of the TA mode are degenerated along the Γ–Z direction. The significant absolute values of Grüneisen parameters uncover the strong phonon anharmonicity, and result in rather low thermal conductivity. The distribution of the mode Grüneisen parameters throughout the first Brillouin zone not only reflects the phonon anharmonicity, but also shows the existence of soft modes for the acoustic phonons. The Grüneisen parameter of the lowest *E* mode is −5.73, which agrees well with recent calculational work [18], suggesting the lowest *E* mode can lead to a structural instability at a low energy. In addition, the averaged Grüneisen parameter $\left(\bar{\gamma }\right)$ can be approximated with the root mean square average of the generalized mode Grüneisen parameter per mode, represented as $\bar{\gamma }=\sqrt{\gamma^{2}}$ [49]. The calculated averaged Grüneisen parameters for all branches along the high-symmetry path at different hydrostatic pressures are shown in Table 2.

The average of the Grüneisen parameters along the path through the center point Γ is larger than that along the X–P path, which reflects the stronger phonon anharmonicity near center point Γ. The increase of the hydrostatic pressure has little effect on the average of the Grüneisen parameters along the X–P path below 4 GPa. The average Grüneisen parameters along the other 3 paths (Z–Γ, Γ–X, N–Γ) accelerate monotonously with pressure, particularly above 3 GPa. The enhancement of phonon anharmonicity makes pressure as a direct and effective way reduce thermal conductivity of chalcopyrite type compound AgGaS_2_ and promotes its application in the field of thermoelectricity. The large average Grüneisen parameters (6.33, 7.86 and 5.67 along the Z-Γ, Γ-X, and P-Γ paths, respectively) at 4 GPa indicate the structural instability may be discovered within this area.

### 3.3. Thermodynamic Properties

Thermodynamic properties are the fundamental basis for the technical application of materials. As a further application, we calculated the thermodynamic properties based on the quasi-harmonic approximation using the phonon frequencies on a sampling mesh in the reciprocal space. Using the quasi-harmonic approximation, the energy at the wave vector ***q*** and band index *j* can be given as:

(6) Eqj=ℏωqj[12+1exp(ℏωqjkBT)−1],

where, $\omega_{qj}$ is the frequency of the *j*th phonon mode at wave vector ***q***; ℏ, $k_{B}$, and *T* are the reduced Plank constant, Boltzmann constant and absolute temperature, respectively. Based on the thermodynamic relations, the constant volume heat capacity $C_{V,qj}$ of the *j*th phonon mode at wave vector ***q*** can be represented as:

(7) $$C_{V,qj}={\left(\frac{\partial E_{qj}}{\partial T}\right)}_{V}.$$

The total constant volume heat capacity $C_{V}$, contributed from all phonon vibration modes in the whole Brillouin zone is given as:

(8) $$C_{V}=\sum_{qj}C_{V,qj}=\sum_{qj}k_{B}{\left(\frac{\hbar \omega_{qj}}{k_{B}T}\right)}^{2}\frac{\exp\left(\hbar \omega_{qj}/k_{B}T\right)}{{\left[\exp{\left(\hbar \omega_{qj}/k_{B}T\right)}^{-1}\right]}^{2}}.$$

According to thermodynamics, the Helmholtz free energy *F*(*V*,*T*) of a solid material can be written as the following formula:

(9) $$F\left(V,T\right)=E_{0}\left(V\right)+F_{eh}\left(V,T\right)+F_{ph}\left(V,T\right),$$

where $E_{0}\left(V\right)$ is the static energy at a constant volume *V* and *T* = 0, which can be obtained from the first-principle calculation. $F_{eh}\left(V,T\right)$ and $F_{ph}\left(V,T\right)$ denote the thermal free energy contribution from the electron and phonon, respectively. Compared with the thermal free energy contribution from the phonon thermal excitation, the contribution from the electron excitation can be ignored; we only considered the phonon contribution in our work. Within the quasi-harmonic approximation, $F_{ph}\left(V,T\right)$ is described as the contribution sum from all the phonon branches and wave vectors in the whole Brillouin zone:

(10) $$F_{ph}\left(V,T\right)=\frac{1}{2}\sum_{qj}\hbar \omega_{qj}+k_{B}T\sum_{qj}\ln\left[1-\exp\left(-\hbar \omega_{qj}/k_{B}T\right)\right].$$

The entropy *S* is defined as the first derivative of the Helmholtz free energy versus temperature:

(11) $$S=\frac{\partial F}{\partial T}=\frac{1}{2T}\sum_{qj}\hbar \omega_{qj}\coth\left(\hbar \omega_{qj}/2k_{B}T\right)-k_{B}\sum_{qj}\ln\left[2\sinh\left(\hbar \omega_{qj}/2k_{B}T\right)\right].$$

**Figure 5:**
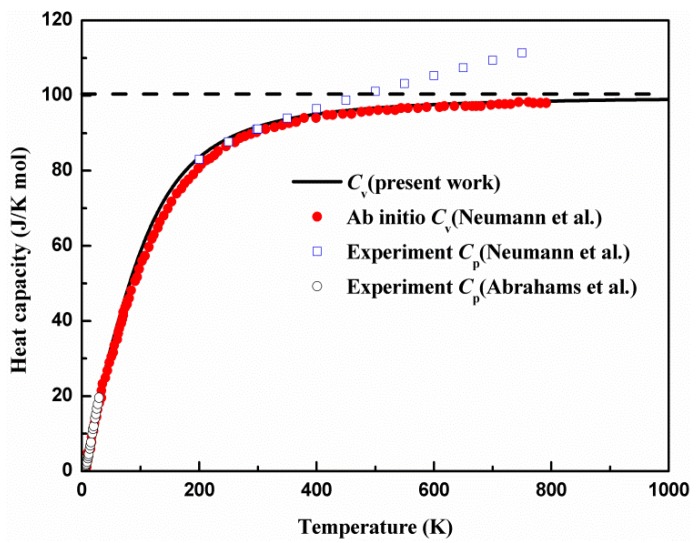
Comparison of theoretical and measured heat capacity of AgGaS_2_ as a function of temperature. The theoretical *C*_v_ data are collected from the ab initio calculation (filled circle) [50]. The measured *C*_p_ (open circle and open square) values are taken from literature [16,51].

The *C*_v_ calculated with the quasi-harmonic approximation is compared with the previous first-principle computation and experimental constant pressure heat capacity *C*_p_ in Figure 5.

The calculational results are in line with previous first-principles theoretical report [50]. The calculated *C*_v_ agrees well with the measured *C*_p_ in the temperature range below 40 K [51]. As the data of the measured *C*_p_ are scarce in the temperature range from 40 to 200 K, it is difficult to compare the calculated data with measured data. We found that the discrepancy between the calculated *C*_v_ and measured *C*_p_ continuously increased with the increase of the temperature for the temperature range above 400 K [16], and this indicates the essential difference between *C*_v_ and *C*_p_. The consistency of the calculated *C*_v_ and measured *C*_p_ at a low temperature implies the contribution from the phonon anharmonicity to the heat capacity is insignificant and can be neglected. The *C*_v_ strongly depends on temperature when temperature is below 200 K. We found that the heat capacity *C*_v_ dramatically increased cubically as a function of temperature at a low temperature (below 200 K). When the temperature is higher than 200 K, the heat capacity rapidly slows with the temperature. The heat capacity almost reaches a constant and follows the Dulong–Petit law at a high temperature (above 700 K), when the anharmonic effect is significant.

**Figure 6:**
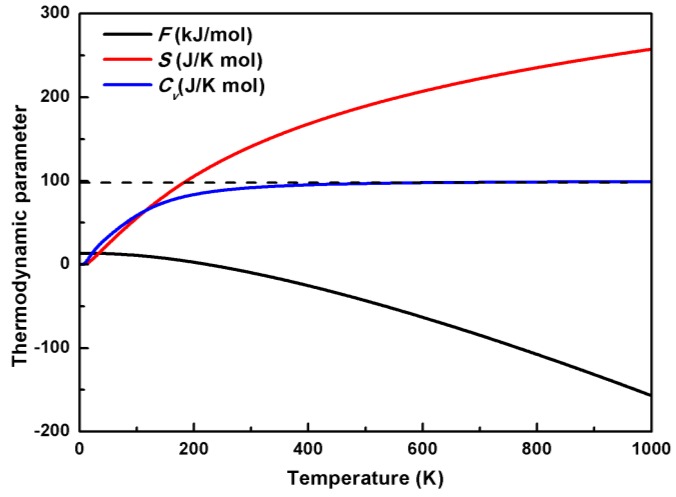
The temperature dependence of the Helmholtz free energy *F*, entropy *S*, and heat capacity *C*_v_ of AgGaS_2_ at 0 pressure.

The calculated thermodynamic properties, including the Helmholtz free energy *F*, entropy *S*, and heat capacity *C*_v_, for AgGaS_2_ at 0 pressure as a function of the temperature, are presented in Figure 6. Figure 6 shows that the Helmholtz free energy decreases monotonously with the increasing temperature. The entropy and heat capacity increase with the increasing temperature from 0 J/K·mol. As the temperature increases, the entropy increases smoothly, while the tendency of the heat capacity can be divided into 2 sections as mentioned above. The effect of a high temperature on the Helmholtz free energy *F* and entropy *S* is more important than that of low temperature. The profiles of the thermodynamic curves are coincident with the early calculated results using the quasi-harmonic Debye model [52]; the discrepancies between them may be caused by the different treatment methods for the phonon data. As the thermodynamic parameters were directly from the phonon data calculated by first principles, this model is more accurate than that from the Debye model.

To further clarify the pressure dependence of thermodynamic properties, the calculated Helmholtz free energy *F*, entropy *S*, and heat capacity *C*_v_ of AgGaS_2_ at different temperatures (from 200 K to 1000 K, with intervals of 200 K) and different pressures (from 0 GPa to 4 GPa with intervals of 1 GPa) are listed in Table 3.

Table 3 shows that the entropy and heat capacity linearly decrease approximately with the increase of the pressure at a constant temperature. The Helmholtz free energy has an opposite trend with the pressure change. The data in Table 3 also indicates that the impact of the temperature on the thermodynamic properties is more significant than that of the pressure. For example, the calculated *S* at 200 K are 105.39 and 102.44 J/K·mol under 0 and 4 GPa, respectively.

## 4. Conclusions

The structural, vibrational and thermodynamic properties of the chalcopyrite-type compound AgGaS_2_ under different hydrostatic pressures were investigated by using the first-principles calculation and quasi-harmonic approximation. The calculated lattice constants before applying pressure are consistent with the previous experimental and theoretical reports. The lattice constants and bond lengths were observed to decrease with increasing pressure, which leads to a higher phonon frequency of the optical branches. By using the phonon dispersion curve analysis at different pressures, we found that the chalcopyrite-type compound AgGaS_2_ is dynamically unstable above 4 GPa. The Grüneisen parameters at different pressures were obtained, and these indicate that AgGaS_2_ has negative coefficient of thermal expansion. The analysis of the vibrational modes and Grüneisen parameters reveals that structural instability is associated with the softening of the *E* vibrational mode at around the center point Γ. Increasing external pressure is a direct and effective method to reduce the thermal conductivity for chalcopyrite-type compound AgGaS_2_. The thermodynamic properties for AgGaS_2_, such as the Helmholtz free energy, entropy, and heat capacity, were analyzed at different temperatures and pressures. The results shown in this work provide guidelines for future single-crystal synthesis, the Raman spectroscopic study, and thermoelectric application of AgGaS_2_ under pressure.

## Figures and Tables

**Table 1:** Calculated frequencies of the optical phonons (THz) at center point Γ of the Brillouin zone.

Irreps	0 GPa			1 GPa	2 GPa	3 GPa	4 GPa
		Reference [19]	Reference [41]				
*A*_1_	8.16	8.88	8.85	8.36	8.51	8.83	9.00
*A*_2_	9.87	10.17		10.07	10.26	10.44	10.60
*B*_1_	7.06	6.84		7.23	7.39	7.55	7.69
	9.29	10.11	10.20	9.51	9.71	9.91	10.09
	5.42	5.76	5.70	5.47	5.52	5.57	5.61
	1.67	1.59	1.62	1.71	1.75	1.78	1.81
*B*_2_ (LO/TO)	10.03/10.00	12.06/11.07	11.94/10.92	10.27/10.23	10.50/10.45	10.74/10.66	10.94/10.85
	6.37/5.96	7.17/6.42	7.14/6.36	6.57/6.11	6.75/6.24	6.94/6.38	7.11/6.50
	1.80/1.80	1.95/1.92	1.92	1.81/1.81	1.84/1.84	1.86/1.86	1.88/1.88
*E* (LO/TO)	10.96/10.61	12.06/11.22	11.88/11.04	11.17/10.83	11.37/11.04	11.57/11.25	11.74/11.43
	9.29/8.91	10.56/9.78	10.47/9.75	9.51/9.12	9.71/9.30	9.91/9.50	10.08/9.66
	6.64/6.53	7.02/6.75	6.96/6.78	6.76/6.57	6.91/6.89	7.10/7.00	7.27/7.12
	4.56/4.50	4.89/4.47	4.80/4.71	4.62/4.57	4.69/4.65	4.76/4.72	4.83/4.79
	2.80/2.77	2.77/2.77	2.85	2.84/2.80	2.87/2.86	2.90/2.89	2.91/2.91
	1.07/1.07	1.07/0.99	1.02	0.98/0.98	0.89/0.89	0.79/0.79	0.67/0.67

**Table 2:** The average of the Grüneisen parameters along the high-symmetry path for AgGaS_2_.

High-Symmetry Path	0 GPa	1 GPa	2 GPa	3 GPa	4 GPa
Z-Γ	1.65	1.76	1.85	2.21	6.33
Γ-X	1.39	1.61	1.73	2.98	7.86
X-P	1.19	1.18	1.15	1.18	1.79
P-Γ	1.73	1.78	1.80	1.83	5.67

**Table 3:** Helmholtz free energy *F* (kJ/mol), entropy *S* (J/K·mol), and heat capacity *C*_v_ (J/K·mol) of AgGaS_2_ at temperature *T* (K) and pressure *P* (GPa).

*T*	*P*	0 GPa	1 GPa	2 GPa	3 GPa	4 GPa
200	*F*	2.60	2.92	3.24	3.55	3.78
	*S*	105.39	104.57	103.68	102.92	102.44
	*C*_v_	83.51	82.94	82.39	81.84	81.35
400	*F*	−25.33	−24.81	−24.28	−23.79	−23.44
	*S*	167.98	166.91	165.78	164.77	164.07
	*C*_v_	95.05	94.86	97.43	94.47	94.30
600	*F*	−63.08	−62.35	−61.58	−60.88	−60.38
	*S*	207.10	205.98	204.79	203.72	202.98
	*C*_v_	97.61	97.52	97.43	97.34	97.25
800	*F*	−107.46	−106.49	−105.49	104.57	−103.92
	*S*	235.33	234.18	232.98	231.89	231.13
	*C*_v_	98.54	98.49	98.44	98.38	98.34
1000	*F*	−156.81	−155.61	−154.36	−153.23	−152.43
	*S*	257.37	256.22	255.00	253.90	253.13
	*C*_v_	98.98	98.95	98.91	98.88	98.85
